# Choroid Melanoma Metastasis to Spine: A Rare Case Report

**DOI:** 10.1155/2016/2732105

**Published:** 2016-02-18

**Authors:** Hiren Mandaliya, Nandini Singh, Sanila George, Mathew George

**Affiliations:** ^1^Medical Oncology, North West Cancer Centre, Tamworth Rural Referral Hospital, Tamworth, NSW 2340, Australia; ^2^University of Newcastle, Tamworth Rural Referral Hospital, Tamworth, NSW 2340, Australia; ^3^Castlereagh Imaging, 201-203 Peel Street, Tamworth, NSW 2340, Australia

## Abstract

Metastatic choroid melanoma is a highly malignant disease with a limited life expectancy. The liver is the most common site for metastasis of uveal melanoma followed by lung, bone, skin, and subcutaneous tissue. Metastasis from choroidal melanoma usually occurs within the first five years of treatment for primary tumours. Metastatic choroid melanoma to the spine/vertebrae is extremely rare. We report the first case of spinal metastasis from choroid melanoma in a 61-year-old man who had been treated for primary ocular melanoma three years earlier with radioactive plaque brachytherapy. Synchronously, at the time of metastasis, he was also diagnosed as having a new primary lung adenocarcinoma as well. The only other case reported on vertebral metastasis from malignant melanoma of choroid in literature in which primary choroid melanoma was enucleated.

## 1. Introduction

Approximately, 95 percent of melanomas around the eye are found in the uvea, and these melanomas have significant differences from cutaneous melanomas regarding molecular pathogenesis and hence their management [1]. Surgery (enucleation or local resection) and radiation therapy (radioactive plaques, charged particle irradiation, or photon-based radiation therapy) are the main modalities of treatment. There is limited evidence on adjuvant therapy and not a standard practice. There are no systemic curative treatments for the majority of patients with metastatic uveal melanoma. In older, unselected series, the prognosis is poor, with reported median survivals ranging from 2 to 12 months [2, 3]. In many cases, the predominant site of metastatic disease for patients with choroid melanoma is the liver, and this has led to the extensive evolution of treatments targeting hepatic disease. Spinal/vertebral metastasis secondary to choroidal melanoma is an infrequent clinical entity. Advances in the treatment of metastatic melanoma using targeted therapy and immunotherapy have led to the prolongation of overall survival for patients with cutaneous melanoma. Some of these approaches are currently being explored for patients with metastatic uveal melanoma. There is only weak evidence that immunotherapy has activity in the treatment of metastatic uveal melanoma.

## 2. Case Presentation

A 61-year-old male presented with a subacute history of lower thoracic back pain for few weeks. The pain was dull, nonradiating, and intermittent in nature that was not responding to analgesia. There was no history of trauma or fall. He has been diagnosed as having left choroid melanoma three years earlier. His first noticed symptoms are imbalance and left eye visual deterioration. Examination of the left funds revealed moderately large partly melanotic and partly amelanotic choroidal melanoma. The melanoma bisected the fovea of the left eye, and it had a base of 15 mm and a thickness of 4.5 mm as assessed by ultrasonography. He was screened for distant metastasis. Blood tests and imaging inform of CT scans were unremarkable at the time. He received brachytherapy using an 18 mm iodine-125 radioactive plaque, which easily covered the base of the melanoma. Subsequently, he has been monitored by an ophthalmologist regularly and a month before this presentation melanoma thickness had reduced to 2.9 mm. The vision was corrected, right eye 6/6 and left eye 6/9. On examination, apart from T8–T12 spinal tenderness, there were no neurological abnormalities detected. His initial CT scan showed a compression fracture of T10 vertebrae with associated retropulsion of the superior fracture fragment of approximately 3.5 mm and a canal stenosis at that level; findings were confirmed on MRI spine. MRI spine also showed disseminated spinal metastasis at various vertebrae without any additional spinal cord compression. He was commenced on steroids and underwent T10 laminectomy and debulking along with T8 to T12 pedicle screw fixation. He was planned for palliative radiotherapy on recovery. Histopathology from T10 vertebral body was suggestive of metastatic melanoma and immunohistochemistry showed strongly positive SOX10, MITF, HMB45, and S100 confirmed metastatic melanoma. BRAF-V600E mutation was not detected. He had an unremarkable postoperative recovery without any neurological sequelae and he was able to mobilise unaided. His full blood counts, liver function tests, renal functions, electrolytes, and PSA were unremarkable; serum lactate dehydrogenase (LDH) levels were 1716 IU/L (normal range: 105–333 IU/L) elevated. Myeloma screening was negative for paraprotein. His staging CT imaging demonstrated a right upper lobe lung spiculated lesion of size 5.6 mm × 3.8 mm × 5.1 mm that was suspicious for further metastases or a new primary lung pathology. There were suspicious lesions in liver on CT scan and were hyperechoic on ultrasound scan consistent with the hemangiomas of the liver, not appearing to have any typical features suggestive of metastatic deposits. He underwent the endobronchial ultrasound (EBUS) guided lung biopsy that was tested positive for a primary lung carcinoma; immunoperoxidase staining was positive for TTF-1, Napsin A, and Cytokeratin AE1/AE3 and there was negative staining for melanoma markers (S100, HMB45, and Melan-A) and other lung primaries (P40 and P63). Our patient was admitted again with spinal cord compression and lower limb paralysis. His general condition deteriorates quickly and collectively we aimed for palliative management as he was suffering from two advance incurable malignancies. Unfortunately, the patient died within couple of weeks.

## 3. Discussion

Choroid melanomas are the most common primary intraocular malignancy in adults [4]. They represent 5% of all melanomas, but, because of the high rate of metastases and poor response to treatment, they account for about 13% of melanoma deaths. Spinal metastasis of choroid malignant melanoma seems to be exceptional since only one case has been previously published [5]. As there is limited data on spinal metastasis from choroid melanoma, treatment in this situation would be similar for any spinal/vertebral metastasis such as spinal precautions, steroids, decompressive neurosurgery with or without spinal fixation, and radiotherapy. In our case, the patient has been on surveillance since treatment for his choroid melanoma with brachytherapy three years earlier which had confirmed tissue diagnosis of metastatic melanoma from uveal origin inform of spinal metastasis. He underwent neurosurgery, awaiting palliative radiotherapy. While investigating for further systemic disease, unfortunately, he was also diagnosed with another malignancy inform of non-small-cell lung cancer. Before making any further plan, he deteriorates and died. Treatment of metastatic melanoma revolutionised in recent time informs of immunotherapy such as checkpoint inhibitors. Observational data suggest that immunotherapy may have activity in metastatic uveal melanoma [6, 7]; however, in phase III trials that established survival benefit with these agents for metastatic cutaneous melanoma, patients with choroid melanoma were excluded [8]. Our patient did not survive to offer any further treatment. As per our knowledge, this has been the only case report in the literature of choroid melanoma metastasis to spine/vertebrae without liver involvement in which the primary choroidal melanoma was treated with radioactive plaque brachytherapy.
